# Crystal structure of *catena*-poly[[(*N*,*N*-diethyl-3-mesitylsulfonyl-1*H*-1,2,4-triazole-1-carboxamide-κ*N* ^1^)silver(I)]-μ-nitrato-κ^3^ *O*,*O*′:*O*]

**DOI:** 10.1107/S2056989016016662

**Published:** 2016-10-21

**Authors:** Hyunjin Park, Eunjin Kwon, Il Yoon, Jineun Kim

**Affiliations:** a Department of Chemistry (BK21 plus) and Research Institute of Natural Sciences, Gyeongsang National University, Jinju 52828, Republic of Korea; b Photodynamic Therapy Research Institute, School of Nanoscience and Engineering, Inje University, 197 Injero, Gimhae, Gyeongnam 50834, Republic of Korea

**Keywords:** crystal structure, cafenstrole, coordination polymer, silver, triazole, herbicide

## Abstract

The reaction of silver nitrate and cafenstrole (*N*,*N*-diethyl-3-mesitylsulfonyl-1*H*-1,2,4-triazole-1-carboxamide), a triazole herbicide, leads to the title coordination polymer, [Ag(NO_3_)(C_16_H_22_N_4_O_3_S)]_*n*_, whose asymmetric unit comprises one cafenstrole ligand mol­ecule, one Ag^I^ atom and one nitrate ion. The Ag^I^ atom, with a distorted trigonal–pyramidal environment, is coordinated by one nitro­gen atom of a triazole ring, two oxygen atoms of a nitrate ion and one oxygen atom of a neighboring nitrate ion. The coordination bonds between silver and oxygen atoms give rise to a one-dimensional (1D) coordination polymer structure along [001]. The dihedral angle between the planes of the triazole and benzene rings is 87.13 (11)°. In the crystal, the coordination polymer is stabilized by C—H⋯O hydrogen bonds and C—H⋯π inter­actions, resulting in a three-dimensional architecture.

## Abstract 2

The asymmetric unit of the title compound comprises one cafenstrole ligand mol­ecule and one silver nitrate ion. The coordination bonds between silver and oxygen atoms allow a continuous one-dimensional coordination polymer structure along [001]. The three-dimensional architecture is stabilized by C—H⋯O hydrogen bonds and C—H⋯π inter­actions

## Chemical context   

Recently, we have reported the crystal structure of the ligand cafenstrole (**L**; Kang *et al.*, 2015 ▸). Cafenstrole is a triazole herbicide and has been used for rice cultivation as an inhibitor of the germination of grass weeds (Takahashi *et al.*, 2001 ▸). Triazole derivatives have been investigated intensively over the years for pharmaceutical and agricultural purposes (Kumar *et al.*, 2013 ▸; Zhang *et al.*, 2014 ▸). It is very likely that triazole–metal inter­actions play a major role in the biological actions of triazole-containing drugs and agricultural chemicals. 1,2,4-Triazole and its derivatives have gained great attention as ligands to transition metals (Haasnoot, 2000 ▸). To understand the inter­actions of triazoles with metals, further research on the structures of triazole–metal compounds is of great necessity. Thus, our attention will be focused on the diversity of the coordination geometries of 1,2,4-triazole complexes with transition metal ions. Herein, we report the reaction of silver nitrate and cafenstrole to produce the title compound, which is a 1D silver(I) coordination polymer.

## Structural commentary   

**Figure 1:**
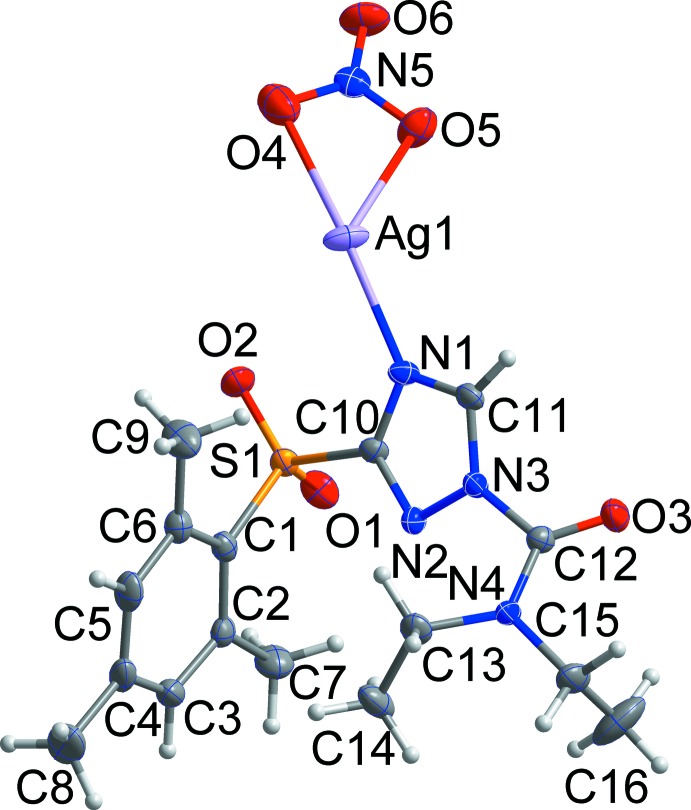
The asymmetric unit of the title compound with the atom-numbering scheme. Displacement ellipsoids are drawn at the 50% probability level. H atoms are shown as small spheres of arbitrary radius.

**Figure 2:**
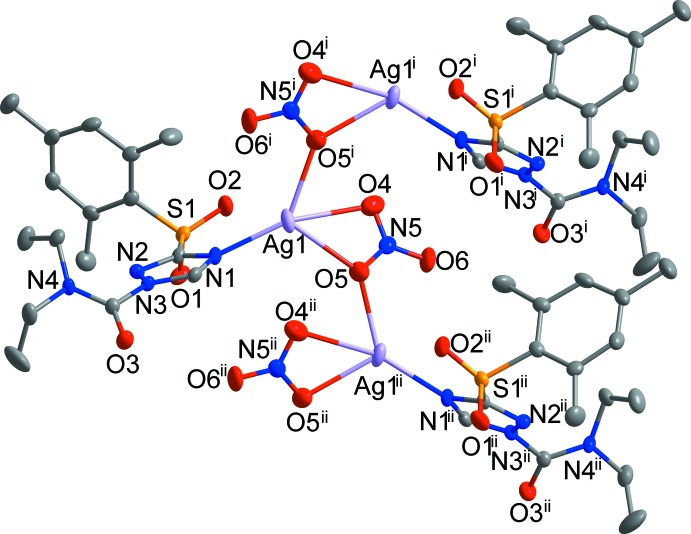
The coordination environment of the Ag^I^ atom in the title compound. [Symmetry codes: (i) −*x* + 1, −*y* + 1, *z* − 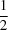; (ii) −*x* + 1, −*y* + 1, *z* + 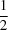.]

The asymmetric unit of the title compound is shown in Fig. 1 ▸. It contains one **L** ligand and one silver nitrate ion. Reaction between silver nitrate and **L** afforded a 1D coordination polymer, in which the Ag^I^ atom has a distorted trigonal–pyramidal environment with one nitro­gen atom (N1) [Ag1—N1 = 2.250 (3) Å] and three oxygen atoms (O4, O5, O5^i^) [Ag1—O4 = 2.708 (3), Ag1—O5 = 2.450 (3) and Ag1—O5^i^ = 2.396 (3) Å; symmetry code: (i) −*x* + 1, −*y* + 1, *z* − 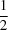], as shown in Fig. 2 ▸. 
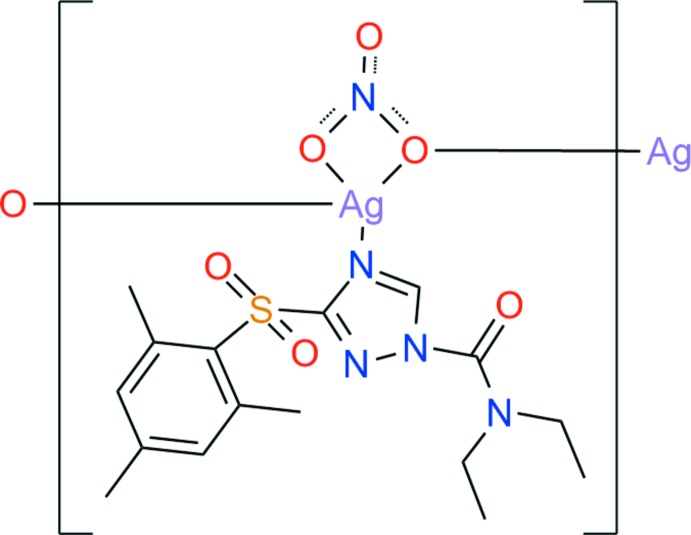


Atom Ag1 lies almost in the plane constituted by atoms O5, N1, and O5^i^ [deviation = 0.0436 (12) Å]. The Ag1, O5, N1, and O5^i^ atoms form a slightly distorted triangular basal plane with bond angles O5—Ag1—O5^i^ = 106.52 (5), O5—Ag1—N1 = 118.75 (11) and O5^i^—Ag1—N1 = 134.63 (11)°. The apex atom, O4, deviates considerably from the normal to the basal plane, as indicated by the O4—Ag1—N1 bond angle of 149.66 (10)°. Other bond angles are 48.93 (10) and 67.18 (10)° for O4—Ag1—O5 and O4—Ag1—O5^i^, respectively. One oxygen atom of the nitrate ion (O6) is not bound to the Ag^I^ ion, whereas the other two oxygen atoms of the nitrate ion (O4 and O5) are bound to the Ag^I^ ion. One of the bound O atoms (O5) links neighbouring Ag^I^ ion ions, thus forming a 1D polymer along [001]. The triazole plane is rotated about the S1–C10 axis in the opposite direction in comparison with free cafenstrol (Kang *et al.*, 2015 ▸). Thus, the diethyl amino group is located above the phenyl ring in the title compound, while that of free cafenstrol is placed outside the phenyl ring.

## Supra­molecular features   

**Figure 3:**
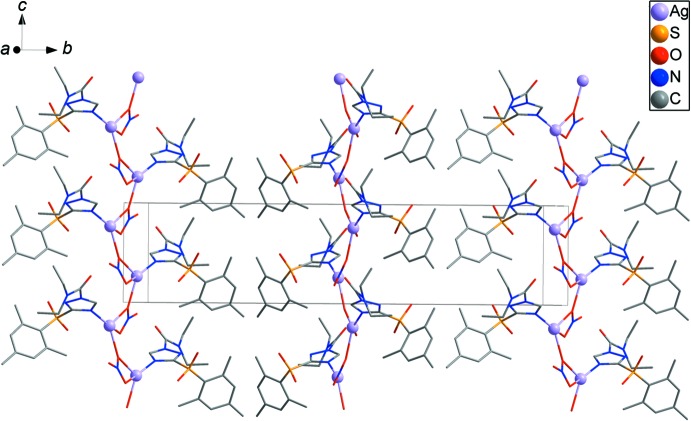
The packing of the title compound showing chains along [001].

**Figure 4:**
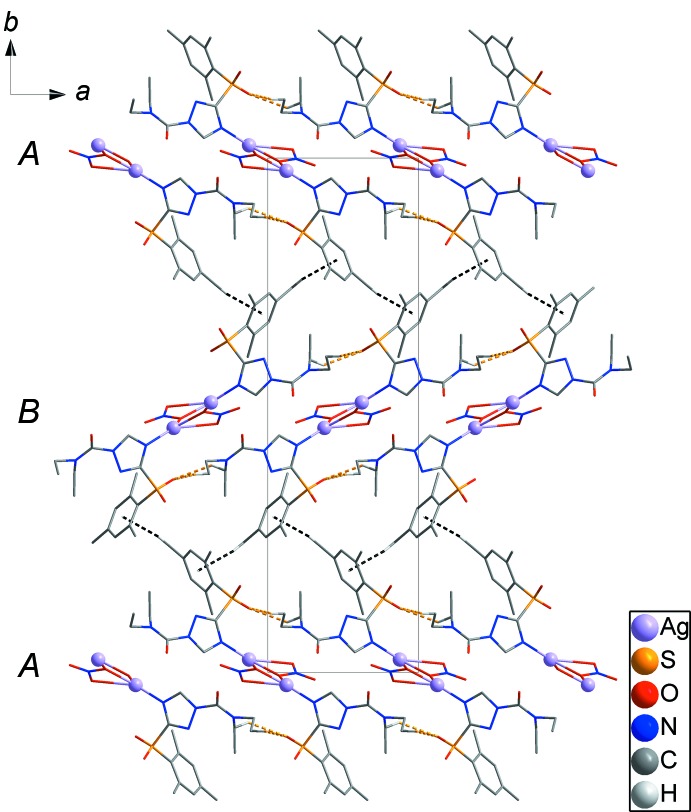
The packing diagram showing the three-dimensional network structure formed by C—H⋯O hydrogen bonds and C—H⋯π inter­actions (shown as yellow and black dashed lines, respectively).

The O5 atom is bound to both Ag1 and neighboring Ag1^ii^ [symmetry code: (ii) −*x* + 1, −*y* + 1, *z* + 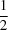], where the neighbouring asymmetric unit is related to the asymmetric unit by 2_1_ symmetry, resulting in a 1D chain along [001] (Fig. 3 ▸). C—H⋯O hydrogen bonds between the 1D chains (yellow dashed lines) lead to the formation of layers parallel to (100). The layers are packed in an *ABAB* pattern along [010] (Fig. 4 ▸). Weak inter­molecular C—H⋯π inter­actions (black dashed lines) between the *A* and *B* layers generate a three-dimensional network structure (Fig. 4 ▸). Thus the structure of the Ag^I^ coordination polymer is stabilized by C13—H13*B*⋯O2 and C16—H16*B*⋯O2 hydrogen bonds and weak inter­molecular C8—H8*C*⋯*Cg*1 (*Cg*1 is the centroid of the C1–C6 ring) inter­actions (Fig. 4 ▸ and Table 1 ▸).

## Database survey   

The crystal structure of cafenstrole has been reported (Kang *et al.*, 2015 ▸). The crystal structure of a 1,2,3-thia­diazole compound containing a 1,2,4-triazole moiety, C_15_H_14_FN_5_O_2_S_2_, has been determined by Min *et al.* (2014 ▸) whereas the structure of a similar triazole herbicide, methyl 2-(1-di­ethyl­carb­amoyl-1,2,4-triazole-3-ylsulfon­yl)acetate, has been reported by Ohkata *et al.* (2002 ▸). The structure of 5-{4-cyclo­propyl-5-[(3-fluoro­benz­yl)sulfin­yl]-4*H*-1,2,4-triazol-3-yl}-4-methyl-1,2,3-thia­diazole (C_15_H_14_FN_5_OS_2_), was determined by Min *et al.* (2015 ▸) and the crystal structure of 1-(mesityl-2-sulfon­yl)-3-nitro-1,2,4-triazole has been determined by Kuroda *et al.* (1982 ▸). The complex, [Pr(C_7_H_5_O_3_)_2_(NO_3_)(C_12_H_8_N_2_)]·2C_12_H_8_N_2_, has a polymeric chain structure, where nitrate ions show similar coordination bonds compared to those in the title compound, but with Ag^I^ ions replaced by with Pr^III^ atoms (Wang *et al.*, 2012 ▸).

## Synthesis and crystallization   

The title compound was prepared from a mixed solution of the cafenstrole ligand (0.05 g, 0.14 mmol) in acetone (5 mL) and Ag(NO_3_) (0.06 g, 0.35 mmol) in methanol (5 mL). The ligand was purchased from the Dr Ehrenstorfer GmbH Company. Single crystals suitable for X-ray crystallography were obtained by slow evaporation of the solvent at room temperature after one week.

## Refinement   

Crystal data, data collection and structure refinement details are summarized in Table 2 ▸. All H atoms were positioned geometrically and refined using a riding model with *d*(C—H) = 0.98 Å, *U*_iso_(H) = 1.5*U*_eq_(C) for methyl group, *d*(C—H) = 0.99 Å, *U*_iso_(H) = 1.2*U*_eq_(C) for C*sp* ^3^—H and *d*(C—H) = 0.95 Å, *U*_iso_(H) = 1.2*U*_eq_(C) for aromatic C—H.

## Supplementary Material

Crystal structure: contains datablock(s) I, New_Global_Publ_Block. DOI: 10.1107/S2056989016016662/vn2117sup1.cif

Structure factors: contains datablock(s) I. DOI: 10.1107/S2056989016016662/vn2117Isup2.hkl

CCDC reference: 1510357

Additional supporting information: crystallographic information; 3D view; checkCIF report

## Figures and Tables

**Table 1:** Hydrogen-bond geometry (Å, °) *Cg*1 is the centroid of the C1–C6 ring.

*D*—H⋯*A*	*D*—H	H⋯*A*	*D*⋯*A*	*D*—H⋯*A*
C8—H8*C*⋯*Cg*1^i^	0.98	2.66	3.614 (5)	166
C13—H13*B*⋯O2^ii^	0.99	2.58	3.395 (4)	140
C16—H16*B*⋯O2^iii^	0.98	2.52	3.412 (6)	152

Symmetry codes: (i) 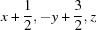; (ii) 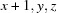; (iii) 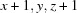.

**Table 2:** Experimental details

Crystal data	
Chemical formula	[Ag(NO_3_)(C_16_H_22_N_4_O_3_S)]
*M*_r_	520.31
Crystal system, space group	Orthorhombic, *P* *n* *a*2_1_
Temperature (K)	173
*a*, *b*, *c* (Å)	9.0947 (2), 31.0133 (6), 7.1934 (1)
*V* (Å^3^)	2028.95 (7)
*Z*	4
Radiation type	Mo *K*α
μ (mm^−1^)	1.14
Crystal size (mm)	0.48 × 0.10 × 0.02

Data collection	
Diffractometer	Bruker APEXII CCD
Absorption correction	Multi-scan (*SADABS*; Bruker, 2014 ▸)
*T*_min_, *T*_max_	0.579, 0.746
No. of measured, independent and observed [*I* > 2σ(*I*)] reflections	17454, 4760, 4259
*R*_int_	0.042
(sin θ/λ)_max_ (Å^−1^)	0.667

Refinement	
*R*[*F* ^2^ > 2σ(*F* ^2^)], *wR*(*F* ^2^), *S*	0.030, 0.053, 0.98
No. of reflections	4760
No. of parameters	267
No. of restraints	1
H-atom treatment	H-atom parameters constrained
Δρ_max_, Δρ_min_ (e Å^−3^)	0.65, −0.39
Absolute structure	Flack *x* determined using 1577 quotients [(*I* ^+^)−(*I* ^−^)]/[(*I* ^+^)+(*I* ^−^)] (Parsons et al., 2013 ▸)
Absolute structure parameter	0.003 (14)

Computer programs: *APEX2* and *SAINT* (Bruker, 2014 ▸), *SHELXS97* and *SHELXTL* (Sheldrick, 2008 ▸), *SHELXL2014* (Sheldrick, 2015 ▸), *DIAMOND* (Brandenburg, 2010 ▸) and *publCIF* (Westrip, 2010 ▸).

## supplementary crystallographic information

### Crystal data

**Table:**

[Ag(C_16_H_22_N_4_O_3_S)(NO_3_)]	*D*_x_ = 1.703 Mg m^−^^3^
*M**_r_* = 520.31	Mo *K*α radiation, λ = 0.71073 Å
Orthorhombic, *P**n**a*2_1_	Cell parameters from 7087 reflections
*a* = 9.0947 (2) Å	θ = 2.3–27.1°
*b* = 31.0133 (6) Å	µ = 1.14 mm^−^^1^
*c* = 7.1934 (1) Å	*T* = 173 K
*V* = 2028.95 (7) Å^3^	Plate, colourless
*Z* = 4	0.48 × 0.10 × 0.02 mm
*F*(000) = 1056	

### Data collection

**Table:**

Bruker APEXII CCD diffractometer	4259 reflections with *I* > 2σ(*I*)
φ and ω scans	*R*_int_ = 0.042
Absorption correction: multi-scan (SADABS; Bruker, 2014)	θ_max_ = 28.3°, θ_min_ = 2.3°
*T*_min_ = 0.579, *T*_max_ = 0.746	*h* = −12→11
17454 measured reflections	*k* = −39→41
4760 independent reflections	*l* = −9→9

### Refinement

**Table:**

Refinement on *F*^2^	Hydrogen site location: inferred from neighbouring sites
Least-squares matrix: full	H-atom parameters constrained
*R*[*F*^2^ > 2σ(*F*^2^)] = 0.030	*w* = 1/[σ^2^(*F*_o_^2^) + (0.0179*P*)^2^] where *P* = (*F*_o_^2^ + 2*F*_c_^2^)/3
*wR*(*F*^2^) = 0.053	(Δ/σ)_max_ = 0.002
*S* = 0.98	Δρ_max_ = 0.65 e Å^−^^3^
4760 reflections	Δρ_min_ = −0.39 e Å^−^^3^
267 parameters	Absolute structure: Flack *x* determined using 1577 quotients [(*I*^+^)-(*I*^-^)]/[(*I*^+^)+(*I*^-^)] (Parsons et al., 2013)
1 restraint	Absolute structure parameter: 0.003 (14)

### Special details

**Table:**

Geometry. All esds (except the esd in the dihedral angle between two l.s. planes) are estimated using the full covariance matrix. The cell esds are taken into account individually in the estimation of esds in distances, angles and torsion angles; correlations between esds in cell parameters are only used when they are defined by crystal symmetry. An approximate (isotropic) treatment of cell esds is used for estimating esds involving l.s. planes.

### Fractional atomic coordinates and isotropic or equivalent isotropic displacement parameters (Å^2^)

**Table:**

	*x*	*y*	*z*	*U*_iso_*/*U*_eq_	
Ag1	0.62392 (3)	0.52477 (2)	0.76161 (6)	0.03314 (9)	
S1	0.73402 (9)	0.64406 (3)	0.83512 (13)	0.01964 (19)	
O1	0.6829 (3)	0.66757 (8)	0.9943 (4)	0.0300 (7)	
O2	0.6265 (2)	0.62494 (9)	0.7166 (4)	0.0309 (8)	
O3	1.1685 (2)	0.53847 (7)	1.2570 (5)	0.0290 (5)	
O4	0.3336 (4)	0.51667 (11)	0.6854 (5)	0.0486 (9)	
O5	0.4227 (3)	0.49348 (10)	0.9434 (4)	0.0382 (7)	
O6	0.1895 (3)	0.48605 (10)	0.8835 (5)	0.0424 (8)	
N1	0.8072 (3)	0.55871 (9)	0.9126 (4)	0.0206 (7)	
N2	0.9749 (3)	0.60970 (9)	0.9892 (4)	0.0187 (6)	
N3	1.0242 (3)	0.56942 (9)	1.0338 (4)	0.0183 (6)	
N4	1.2736 (3)	0.58767 (9)	1.0626 (4)	0.0206 (7)	
N5	0.3120 (4)	0.49853 (10)	0.8351 (5)	0.0266 (8)	
C1	0.8614 (3)	0.67468 (11)	0.7068 (5)	0.0205 (9)	
C2	0.9148 (3)	0.71461 (10)	0.7730 (7)	0.0218 (7)	
C3	1.0110 (4)	0.73701 (13)	0.6569 (6)	0.0286 (9)	
H3	1.0465	0.7642	0.6973	0.034*	
C4	1.0571 (4)	0.72185 (14)	0.4873 (6)	0.0300 (9)	
C5	1.0073 (4)	0.68155 (14)	0.4304 (6)	0.0303 (10)	
H5	1.0412	0.6703	0.3152	0.036*	
C6	0.9102 (4)	0.65722 (12)	0.5352 (5)	0.0232 (8)	
C7	0.8791 (4)	0.73432 (14)	0.9579 (6)	0.0353 (10)	
H7A	0.9450	0.7587	0.9811	0.053*	
H7B	0.8919	0.7127	1.0559	0.053*	
H7C	0.7769	0.7444	0.9576	0.053*	
C8	1.1603 (5)	0.74778 (18)	0.3669 (7)	0.0526 (14)	
H8A	1.1032	0.7677	0.2896	0.079*	
H8B	1.2166	0.7283	0.2868	0.079*	
H8C	1.2279	0.7642	0.4459	0.079*	
C9	0.8679 (5)	0.61345 (14)	0.4610 (6)	0.0391 (11)	
H9A	0.7697	0.6150	0.4054	0.059*	
H9B	0.8673	0.5925	0.5630	0.059*	
H9C	0.9391	0.6044	0.3665	0.059*	
C10	0.8450 (3)	0.60100 (11)	0.9190 (5)	0.0177 (8)	
C11	0.9224 (4)	0.53946 (12)	0.9906 (5)	0.0216 (8)	
H11	0.9315	0.5094	1.0124	0.026*	
C12	1.1648 (4)	0.56330 (12)	1.1294 (5)	0.0193 (8)	
C13	1.2803 (4)	0.60769 (12)	0.8765 (6)	0.0254 (9)	
H13A	1.1939	0.5983	0.8031	0.030*	
H13B	1.3697	0.5974	0.8116	0.030*	
C14	1.2828 (4)	0.65612 (13)	0.8847 (7)	0.0365 (11)	
H14A	1.1910	0.6666	0.9400	0.055*	
H14B	1.2929	0.6678	0.7587	0.055*	
H14C	1.3661	0.6656	0.9608	0.055*	
C15	1.4092 (4)	0.58914 (14)	1.1768 (6)	0.0319 (10)	
H15A	1.4890	0.6026	1.1035	0.038*	
H15B	1.4398	0.5593	1.2073	0.038*	
C16	1.3886 (5)	0.6139 (2)	1.3531 (8)	0.0642 (17)	
H16A	1.3571	0.6433	1.3238	0.096*	
H16B	1.4816	0.6148	1.4217	0.096*	
H16C	1.3134	0.5998	1.4294	0.096*	

### Atomic displacement parameters (Å^2^)

**Table:**

	*U*^11^	*U*^22^	*U*^33^	*U*^12^	*U*^13^	*U*^23^
Ag1	0.01833 (12)	0.03174 (15)	0.04934 (19)	−0.00517 (11)	−0.00399 (18)	−0.01117 (19)
S1	0.0136 (4)	0.0221 (4)	0.0232 (5)	0.0013 (3)	0.0017 (4)	0.0027 (4)
O1	0.0276 (14)	0.0292 (15)	0.0333 (18)	0.0050 (11)	0.0131 (13)	0.0002 (13)
O2	0.0178 (11)	0.0376 (15)	0.037 (2)	−0.0045 (10)	−0.0073 (12)	0.0054 (13)
O3	0.0248 (12)	0.0308 (13)	0.0315 (14)	−0.0015 (9)	−0.0036 (18)	0.0114 (18)
O4	0.064 (2)	0.044 (2)	0.037 (2)	−0.0106 (17)	0.0084 (16)	0.0004 (17)
O5	0.0227 (13)	0.057 (2)	0.0346 (19)	0.0013 (14)	−0.0001 (14)	−0.0022 (16)
O6	0.0200 (14)	0.053 (2)	0.054 (2)	−0.0094 (13)	0.0092 (14)	−0.0063 (16)
N1	0.0154 (14)	0.0192 (16)	0.0272 (19)	−0.0024 (12)	0.0000 (13)	−0.0001 (14)
N2	0.0189 (14)	0.0181 (15)	0.0192 (17)	0.0011 (11)	−0.0022 (13)	0.0017 (13)
N3	0.0164 (14)	0.0183 (16)	0.0203 (17)	−0.0009 (11)	−0.0002 (13)	0.0040 (14)
N4	0.0152 (14)	0.0212 (16)	0.0253 (19)	−0.0034 (12)	−0.0062 (13)	0.0060 (14)
N5	0.0290 (18)	0.0226 (18)	0.028 (2)	−0.0009 (14)	0.0059 (16)	−0.0059 (16)
C1	0.0175 (16)	0.0221 (18)	0.022 (2)	0.0025 (13)	0.0002 (15)	0.0049 (14)
C2	0.0220 (15)	0.0231 (16)	0.0204 (19)	0.0025 (12)	−0.006 (2)	0.003 (2)
C3	0.029 (2)	0.028 (2)	0.029 (2)	−0.0089 (17)	−0.0079 (18)	0.0088 (19)
C4	0.0210 (19)	0.045 (3)	0.024 (2)	−0.0072 (17)	−0.0035 (18)	0.013 (2)
C5	0.026 (2)	0.045 (3)	0.019 (2)	0.0022 (18)	0.0054 (18)	0.0061 (19)
C6	0.0224 (18)	0.026 (2)	0.021 (2)	0.0042 (15)	0.0016 (16)	0.0023 (17)
C7	0.044 (2)	0.025 (2)	0.037 (3)	−0.0028 (18)	−0.001 (2)	−0.0045 (19)
C8	0.043 (3)	0.078 (4)	0.037 (3)	−0.026 (3)	0.002 (2)	0.019 (3)
C9	0.059 (3)	0.031 (2)	0.027 (3)	−0.004 (2)	0.008 (2)	−0.008 (2)
C10	0.0126 (16)	0.0185 (18)	0.022 (2)	0.0003 (13)	0.0030 (15)	0.0006 (16)
C11	0.0222 (18)	0.0211 (18)	0.021 (2)	−0.0047 (14)	0.0032 (17)	0.0029 (16)
C12	0.0166 (17)	0.0185 (19)	0.023 (2)	0.0010 (13)	0.0000 (16)	0.0005 (16)
C13	0.0181 (17)	0.031 (2)	0.027 (2)	−0.0019 (15)	0.0039 (16)	0.0053 (17)
C14	0.030 (2)	0.027 (2)	0.052 (3)	−0.0036 (17)	0.000 (2)	0.016 (2)
C15	0.0199 (19)	0.033 (2)	0.042 (3)	−0.0039 (17)	−0.0091 (18)	0.007 (2)
C16	0.045 (3)	0.095 (5)	0.053 (3)	−0.001 (3)	−0.026 (3)	−0.025 (3)

### Geometric parameters (Å, º)

**Table:**

Ag1—N1	2.250 (3)	C4—C5	1.391 (6)
Ag1—O5^i^	2.396 (3)	C4—C8	1.509 (5)
Ag1—O5	2.450 (3)	C5—C6	1.385 (5)
S1—O2	1.426 (3)	C5—H5	0.9500
S1—O1	1.435 (3)	C6—C9	1.509 (5)
S1—C1	1.760 (4)	C7—H7A	0.9800
S1—C10	1.779 (3)	C7—H7B	0.9800
O3—C12	1.198 (5)	C7—H7C	0.9800
O4—N5	1.231 (4)	C8—H8A	0.9800
O5—N5	1.282 (4)	C8—H8B	0.9800
O5—Ag1^ii^	2.396 (3)	C8—H8C	0.9800
O6—N5	1.230 (4)	C9—H9A	0.9800
N1—C11	1.330 (5)	C9—H9B	0.9800
N1—C10	1.357 (4)	C9—H9C	0.9800
N2—C10	1.313 (4)	C11—H11	0.9500
N2—N3	1.365 (4)	C13—C14	1.503 (5)
N3—C11	1.348 (4)	C13—H13A	0.9900
N3—C12	1.465 (4)	C13—H13B	0.9900
N4—C12	1.334 (4)	C14—H14A	0.9800
N4—C13	1.477 (5)	C14—H14B	0.9800
N4—C15	1.483 (5)	C14—H14C	0.9800
C1—C2	1.413 (5)	C15—C16	1.494 (6)
C1—C6	1.419 (5)	C15—H15A	0.9900
C2—C3	1.395 (5)	C15—H15B	0.9900
C2—C7	1.499 (7)	C16—H16A	0.9800
C3—C4	1.373 (6)	C16—H16B	0.9800
C3—H3	0.9500	C16—H16C	0.9800
			
N1—Ag1—O5^i^	134.63 (11)	H7A—C7—H7C	109.5
N1—Ag1—O5	118.75 (11)	H7B—C7—H7C	109.5
O5^i^—Ag1—O5	106.52 (5)	C4—C8—H8A	109.5
O2—S1—O1	117.78 (16)	C4—C8—H8B	109.5
O2—S1—C1	111.23 (17)	H8A—C8—H8B	109.5
O1—S1—C1	110.96 (16)	C4—C8—H8C	109.5
O2—S1—C10	106.22 (17)	H8A—C8—H8C	109.5
O1—S1—C10	107.14 (17)	H8B—C8—H8C	109.5
C1—S1—C10	102.10 (16)	C6—C9—H9A	109.5
N5—O5—Ag1^ii^	118.1 (2)	C6—C9—H9B	109.5
N5—O5—Ag1	102.3 (2)	H9A—C9—H9B	109.5
Ag1^ii^—O5—Ag1	137.37 (12)	C6—C9—H9C	109.5
C11—N1—C10	102.7 (3)	H9A—C9—H9C	109.5
C11—N1—Ag1	125.2 (2)	H9B—C9—H9C	109.5
C10—N1—Ag1	131.0 (2)	N2—C10—N1	116.1 (3)
C10—N2—N3	101.4 (3)	N2—C10—S1	119.1 (3)
C11—N3—N2	110.5 (3)	N1—C10—S1	124.8 (3)
C11—N3—C12	128.2 (3)	N1—C11—N3	109.2 (3)
N2—N3—C12	121.0 (3)	N1—C11—H11	125.4
C12—N4—C13	126.5 (3)	N3—C11—H11	125.4
C12—N4—C15	115.7 (3)	O3—C12—N4	128.3 (3)
C13—N4—C15	117.1 (3)	O3—C12—N3	117.9 (3)
O6—N5—O4	122.4 (4)	N4—C12—N3	113.9 (3)
O6—N5—O5	120.0 (4)	N4—C13—C14	112.6 (3)
O4—N5—O5	117.6 (4)	N4—C13—H13A	109.1
C2—C1—C6	121.3 (3)	C14—C13—H13A	109.1
C2—C1—S1	121.5 (3)	N4—C13—H13B	109.1
C6—C1—S1	117.2 (3)	C14—C13—H13B	109.1
C3—C2—C1	116.8 (4)	H13A—C13—H13B	107.8
C3—C2—C7	117.6 (3)	C13—C14—H14A	109.5
C1—C2—C7	125.6 (3)	C13—C14—H14B	109.5
C4—C3—C2	123.6 (4)	H14A—C14—H14B	109.5
C4—C3—H3	118.2	C13—C14—H14C	109.5
C2—C3—H3	118.2	H14A—C14—H14C	109.5
C3—C4—C5	118.0 (4)	H14B—C14—H14C	109.5
C3—C4—C8	121.2 (4)	N4—C15—C16	112.4 (4)
C5—C4—C8	120.8 (4)	N4—C15—H15A	109.1
C6—C5—C4	122.5 (4)	C16—C15—H15A	109.1
C6—C5—H5	118.8	N4—C15—H15B	109.1
C4—C5—H5	118.8	C16—C15—H15B	109.1
C5—C6—C1	117.7 (4)	H15A—C15—H15B	107.9
C5—C6—C9	117.4 (4)	C15—C16—H16A	109.5
C1—C6—C9	124.8 (3)	C15—C16—H16B	109.5
C2—C7—H7A	109.5	H16A—C16—H16B	109.5
C2—C7—H7B	109.5	C15—C16—H16C	109.5
H7A—C7—H7B	109.5	H16A—C16—H16C	109.5
C2—C7—H7C	109.5	H16B—C16—H16C	109.5
			
C10—N2—N3—C11	−0.7 (4)	N3—N2—C10—N1	−0.7 (4)
C10—N2—N3—C12	−176.0 (3)	N3—N2—C10—S1	−179.2 (2)
Ag1^ii^—O5—N5—O6	−14.1 (4)	C11—N1—C10—N2	1.8 (4)
Ag1—O5—N5—O6	179.9 (3)	Ag1—N1—C10—N2	−166.7 (3)
Ag1^ii^—O5—N5—O4	164.7 (3)	C11—N1—C10—S1	−179.8 (3)
Ag1—O5—N5—O4	−1.3 (4)	Ag1—N1—C10—S1	11.7 (5)
O2—S1—C1—C2	140.4 (3)	O2—S1—C10—N2	161.7 (3)
O1—S1—C1—C2	7.2 (3)	O1—S1—C10—N2	−71.6 (3)
C10—S1—C1—C2	−106.7 (3)	C1—S1—C10—N2	45.1 (3)
O2—S1—C1—C6	−40.6 (3)	O2—S1—C10—N1	−16.7 (4)
O1—S1—C1—C6	−173.8 (3)	O1—S1—C10—N1	110.0 (3)
C10—S1—C1—C6	72.3 (3)	C1—S1—C10—N1	−133.3 (3)
C6—C1—C2—C3	3.5 (5)	C10—N1—C11—N3	−2.1 (4)
S1—C1—C2—C3	−177.6 (3)	Ag1—N1—C11—N3	167.3 (2)
C6—C1—C2—C7	−175.3 (3)	N2—N3—C11—N1	1.9 (4)
S1—C1—C2—C7	3.6 (5)	C12—N3—C11—N1	176.8 (3)
C1—C2—C3—C4	−1.4 (5)	C13—N4—C12—O3	160.4 (4)
C7—C2—C3—C4	177.5 (4)	C15—N4—C12—O3	−10.0 (6)
C2—C3—C4—C5	−1.3 (6)	C13—N4—C12—N3	−21.2 (5)
C2—C3—C4—C8	179.4 (4)	C15—N4—C12—N3	168.4 (3)
C3—C4—C5—C6	2.1 (6)	C11—N3—C12—O3	−39.4 (5)
C8—C4—C5—C6	−178.6 (4)	N2—N3—C12—O3	135.0 (4)
C4—C5—C6—C1	−0.1 (6)	C11—N3—C12—N4	142.0 (4)
C4—C5—C6—C9	−178.3 (4)	N2—N3—C12—N4	−43.6 (4)
C2—C1—C6—C5	−2.8 (5)	C12—N4—C13—C14	116.8 (4)
S1—C1—C6—C5	178.2 (3)	C15—N4—C13—C14	−73.0 (4)
C2—C1—C6—C9	175.2 (4)	C12—N4—C15—C16	−70.8 (5)
S1—C1—C6—C9	−3.8 (5)	C13—N4—C15—C16	117.9 (4)

Symmetry codes: (i) −*x*+1, −*y*+1, *z*−1/2; (ii) −*x*+1, −*y*+1, *z*+1/2.

### Hydrogen-bond geometry (Å, º)

Cg1 is the centroid of the C1–C6 ring.

**Table:**

*D*—H···*A*	*D*—H	H···*A*	*D*···*A*	*D*—H···*A*
C8—H8*C*···*Cg*1^iii^	0.98	2.66	3.614 (5)	166
C13—H13*B*···O2^iv^	0.99	2.58	3.395 (4)	140
C16—H16*B*···O2^v^	0.98	2.52	3.412 (6)	152

Symmetry codes: (iii) *x*+1/2, −*y*+3/2, *z*; (iv) *x*+1, *y*, *z*; (v) *x*+1, *y*, *z*+1.
